# Bilinear Distance Feature Network for Semantic Segmentation in PowerLine Corridor Point Clouds

**DOI:** 10.3390/s24155021

**Published:** 2024-08-02

**Authors:** Yunyi Zhou, Ziyi Feng, Chunling Chen, Fenghua Yu

**Affiliations:** 1 College of Information and Electrical Engineering, Shenyang Agricultural University, Shenyang 110866, China; 2022220018@stu.syau.edu.cn (Y.Z.); adan@syau.edu.cn (F.Y.); 2 National Digital Agriculture Regional Innovation Center (Northeast), Shenyang 110866, China; 3 Key Laboratory of Smart Agriculture Technology in Liaoning Province, Shenyang 110866, China

**Keywords:** powerline corridor, 3D point clouds, semantic segmentation, feature extraction

## Abstract

Semantic segmentation of target objects in power transmission line corridor point cloud scenes is a crucial step in powerline tree barrier detection. The massive quantity, disordered distribution, and non-uniformity of point clouds in power transmission line corridor scenes pose significant challenges for feature extraction. Previous studies have often overlooked the core utilization of spatial information, limiting the network’s ability to understand complex geometric shapes. To overcome this limitation, this paper focuses on enhancing the deep expression of spatial geometric information in segmentation networks and proposes a method called BDF-Net to improve RandLA-Net. For each input 3D point cloud data, BDF-Net first encodes the relative coordinates and relative distance information into spatial geometric feature representations through the Spatial Information Encoding block to capture the local spatial structure of the point cloud data. Subsequently, the Bilinear Pooling block effectively combines the feature information of the point cloud with the spatial geometric representation by leveraging its bilinear interaction capability thus learning more discriminative local feature descriptors. The Global Feature Extraction block captures the global structure information in the point cloud data by using the ratio between the point position and the relative position, so as to enhance the semantic understanding ability of the network. In order to verify the performance of BDF-Net, this paper constructs a dataset, PPCD, for the point cloud scenario of transmission line corridors and conducts detailed experiments on it. The experimental results show that BDF-Net achieves significant performance improvements in various evaluation metrics, specifically achieving an OA of 97.16%, a mIoU of 77.48%, and a mAcc of 87.6%, which are 3.03%, 16.23%, and 18.44% higher than RandLA-Net, respectively. Moreover, comparisons with other state-of-the-art methods also verify the superiority of BDF-Net in point cloud semantic segmentation tasks.

## 1. Introduction

The power industry, as a crucial pillar industry affecting the lives of people across the country, is essential for ensuring its safe, stable, and reliable development. The power transmission line corridor, as a core component of the power system, represents critical infrastructure. When transmission line corridors are located within forests, tree barrier incidents can occur if the height of the trees and the shortest distance between the transmission line sag is less than the safety distance required by power safety regulations. Such incidents can lead to large-scale power outages or forest fires, resulting in significant property damage and ecological destruction [1]. To ensure a stable power supply, the power sector needs to conduct periodic inspections of transmission lines and the surrounding vegetation to prevent tree barrier issues.

Traditional power inspection methods are primarily manual, which are costly and inefficient, making it difficult to meet the growing demands of the power grid. In recent years, with the development of geographic surveying technology, drone-based inspections equipped with optical cameras, infrared cameras, and LiDAR have become the mainstream approach for power inspections. Compared to the limitations of two-dimensional image data in spatial information [2], point cloud data collected by airborne LiDAR can directly obtain the three-dimensional coordinates, color, and other information of target objects, which is highly beneficial for achieving 3D visualization of power transmission line corridor scenes, extracting power lines, and measuring the distance between lines and tree barriers. Therefore, airborne LiDAR has gained widespread application in the field of power inspection.

**Figure 1:**
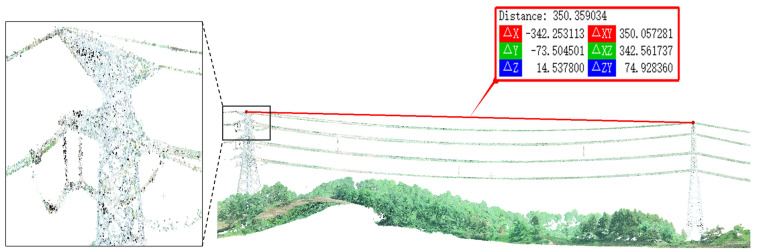
Power transmission line corridor scenes. The illustration (**right**) shows a power transmission line corridor scene for a span, with a horizontal distance of 350.36 m between two towers. The illustration (**left**) is a detailed magnified view of the connection between the transmission line and the tower.

Tree barrier detection for transmission lines includes three parts, namely semantic segmentation of point clouds in power transmission line corridor scenes, extraction and sag prediction of power lines, and measurement of the distance between transmission line sag and vegetation. Among these, precise semantic segmentation of power transmission line corridor scenes is a crucial technical foundation for power line extraction and distance measurement between lines and tree barriers. It facilitates the fine extraction and reconstruction of power line point clouds, and improving its segmentation accuracy is of significant importance for addressing tree barrier issues [3]. The power transmission line corridor scene is shown in Figure 1. Point cloud semantic segmentation refers to the automatic labeling and classification of point clouds in the scene according to their respective features [4]. Unlike image data arranged on a regular pixel grid, point cloud data are a collection of points in a three-dimensional coordinate system that describes the 3D coordinates, depth, color, and other information of objects. This gives point cloud data its characteristics of irregularity and disorder, posing certain challenges for research. Based on the differences in the processing methods of point cloud semantic segmentation models, current point cloud semantic segmentation methods can be divided into two categories— indirect segmentation and direct segmentation [5].

Indirect segmentation methods refer to transforming point cloud data into structured and ordered views or grids for processing and segmentation. Su et al. [6] proposed the Multi-view CNN, which projects 3D objects into multiple 2D views. CNN operations are applied to these 2D views to obtain multiple view features, which are then pooled into a global feature for segmentation processing. However, this approach overlooks the spatial structure relationships of the point cloud. PointGrid [7] combines point clouds with grids, using higher-order local approximation functions to represent the geometric information of point clouds within grid cells. This information is then fed into a 3DCNN to extract point cloud features layer by layer. However, these operations are not very effective for scene segmentation. Cylinder et al. [8] proposed using cylindrical partitioning for voxel division to improve the efficiency of feature extraction and designed an asymmetric 3D convolution module to enhance the contextual response of point clouds in both horizontal and vertical directions. However, the aforementioned indirect segmentation methods are easily affected by the distribution of point clouds and the size of the scene, and the data processing procedure is very complex with low computational efficiency. Therefore, some researchers have proposed direct segmentation methods for point cloud data.

Direct segmentation methods refer to processing each point cloud using convolution, multi-layer perceptrons, and other techniques to achieve end-to-end feature learning. Charles et al. proposed the pioneering work for directly processing point cloud data, PointNet [9]; taking advantage of the unordered nature of point clouds, multi-layer perceptrons and max pooling functions are used to extract features. However, the extraction of local features from point clouds is somewhat lacking. To address this issue, the team subsequently proposed PointNet++ [10], using the K-nearest neighbors algorithm to enhance the neighborhood features of each point, a multi-scale grouping method is designed to adaptively aggregate local feature information of points. This approach can increase the receptive field, but it still has limitations in extracting contextual information from point clouds. In recent years, Qian et al. [11] discovered that PointNet++, as a classic point cloud semantic segmentation network, still has significant untapped potential. Consequently, they proposed the PointNeXt model, which improves training strategies using techniques such as color drop, height augmentation, and data scaling. They also introduced inverse residual bottlenecks and separable MLP to effectively expand the model’s depth and breadth. With the widespread application of transformers in image vision detection, Zhao et al. introduced transformers into the 3D point cloud domain and proposed the Point Transformer [12], by using a self-attention network combined with point cloud position information encoding to construct a feature extraction network, surprisingly effective results were achieved. However, this approach results in significant computational load and memory consumption, leading to very low computational efficiency. Lai et al. proposed the Stratified Transformer [13], which employs a hierarchical approach, performing dense sampling on nearby points and sparse sampling on distant points, capturing remote contextual information to enhance the model’s generalization ability. However, its computational cost is high, making it less effective at capturing edge structures. Additionally, it is more suited for indoor scene segmentation and does not perform well in segmentation tasks for large outdoor point cloud scenes. Researchers have proposed several methods for segmenting large outdoor point cloud scenes. For instance, Q et al. proposed RandLA-Net [14], which applied random sampling to large point cloud scenes, which significantly reduced computational costs. Additionally, by utilizing the relative position and distance information of each point, he was able to better learn the geometric structure of the point cloud, compensating for the loss of local feature information. However, there is still considerable room for improvement in its ability to learn spatial contextual features. BBAF-Net [15] utilizes geometric features and semantic features in a bilateral structure to enhance the local contextual information of the point cloud, adaptively fusing them into output features. Weng et al. [16] uses a plane separation network and a plane relationship network to explore the differences between points near the plane and plane points, as well as the relationships between multiple planes. However, the aforementioned methods only consider the geometric features of point clouds and do not sufficiently extract boundary features between different objects. The key point for segmenting power transmission line corridor scenes is to distinguish between power lines and towers. This requires not only studying the geometric relationships of different target objects but also discussing their spatial structural correlations and enhancing the ability to fuse and extract different feature information.

Based on the above research, this paper aims to propose a semantic segmentation method for power transmission line corridor scenes using airborne LiDAR. The main contributions of this paper are as follows:

1. Firstly, considering the spatial geometric characteristics of power transmission line corridor scenes, a spatial information encoding module is proposed. This module encodes the relative coordinate information and relative distance information of the point cloud, effectively extracting the feature information of target objects.
2. Secondly, to enhance feature fusion and model local feature interactions, a bilinear pooling module is proposed. This module deepens feature representation by performing a Fast Fourier Transform on the point cloud vectors.
3. Lastly, to enhance the spatial contextual information of point clouds, a global feature extraction module is introduced. This module gradually increases the receptive field of each point, further improving the segmentation accuracy of the network. The experimental results demonstrate that this approach outperforms current mainstream semantic segmentation algorithms.

## 2. Related Work

### 2.1. Methods for Extracting Point Cloud Features

The point cloud feature extraction module is a crucial component of the three-dimensional semantic segmentation network, particularly the ability to learn features from local regions. For example, PointNet++ [10] uses the Ball query to find all neighboring points within a fixed radius centered at the centroid point. Then, it converts the coordinates of the points within the neighborhood to local coordinates relative to the centroid point, capturing local region features using a method that combines relative coordinates and point features. RS-CNN [17] addresses the irregularity of point clouds and the limitations of classical CNNs in weight sharing, by proposing to learn the Euclidean distance between a single point cloud and its neighboring points. It then encodes their spatial layout to obtain local features of the point cloud. RandLA-Net [14] utilizes neighborhood point features to calculate relative coordinates and Euclidean distances between point clouds for encoding and aggregation, obtaining local features of the point cloud. SCF-Net [18] focuses on the rotation-invariant characteristics of point clouds, choosing to use polar coordinates to represent the local context features of point clouds, reducing the direction sensitivity and enhancing the model’s generalization ability. MSIDA-Net [19] comprehensively considers the spatial information encoding of point clouds, including Cartesian coordinates, spherical coordinates, and cylindrical coordinates, to fully describe the spatial information of point cloud data. Weng et al. [16] noticed the impact of geometric features of planar objects on segmentation results, proposing a nine-dimensional feature representation including normal, centroid, and normal similarity, to encode the overall scene context. FG-Net [20] believes that the inner product of the central point and its neighboring points can evaluate the spatial features of the point, which can capture local contextual relationships and enhance the feature extraction capability by increasing feature similarity.

However, existing point cloud feature extraction methods still have many shortcomings in segmenting boundary details. First, some works [10,17] have not fully utilized point cloud information, only performing geometric feature spatial embedding, lacking the exploration of depth information. Second, some methods [14,16,19] have overly complex information processing, and the superposition of multi-source information does not significantly improve segmentation accuracy. This paper proposes utilizing two-point features—coordinate features and distance features—and enhancing feature expression through their correlation, which is one of the key research points of this paper.

### 2.2. The Use of Pooling Modules in Deep Learning

The pooling module is widely used in point cloud semantic segmentation tasks for feature fusion. It enhances the network’s feature representation by selecting the appropriate learning functions within a fixed-sized window, thereby expanding the receptive field and optimizing network performance. In the field of deep learning, common pooling modules include max pooling, average pooling, mixed pooling, and attention pooling. Max pooling refers to adaptively constructing the size of the dataset using a random factor. Multi-view CNN [6] utilizes max pooling to aggregate feature information from multiple views to achieve the goal of recognizing 3D shapes. Similarly, PointNet [9] also uses max pooling to fuse the deep features of point clouds, achieving point cloud classification and segmentation. However, selecting only the maximum value as the feature representation can lead to the loss of some information in the original data and reduce the accuracy of the elements. Mean pooling divides the input set into multiple regions and calculates the average value of the features in each subregion. Gardner et al. [21] use average pooling to fuse multiple depth feature sets encoded from input images. Similarly, Theodoridis et al. [22] extended real-valued image features using Zernike moments and then used average pooling for global or regional feature fusion. However, this method still only captures partial information, leading to the neglect of other valuable features. Mixed pooling calculates both the maximum value and the average value in each pooling window of the input feature map, and then combines the maximum value and the average value using certain weights or simply a proportional addition to obtain the final pooling result. LPFE-Net [23] captures the maximum local features and local context features through mixed pooling operations. However, mixed pooling cannot eliminate the problem of partial feature information loss and increases sensitivity to data noise. Attention pooling [24] adjusts the size and stride of pooling based on the attention distribution of the input features, enhancing the model’s recognition accuracy by focusing more on key areas. RandLA-Net [14] uses attention pooling to selectively aggregate the position features and neighboring point features of point clouds. Similarly, Wang et al. [25] used attention convolution pooling to discover more useful information related to the current output in the input data, aggregating feature information from multiple views.

However, considering the spatial geometric distribution characteristics of the power transmission line corridor point cloud scene, this paper is inspired by the bilinear model [26] and designs a Bilinear Pooling module for feature vector fusion. This enhances the feature representation capability, expands the depth information, and reduces the model’s sensitivity to parameters, which is also one of the key research points of this paper.

## 3. Our Methods

**Figure 2:**
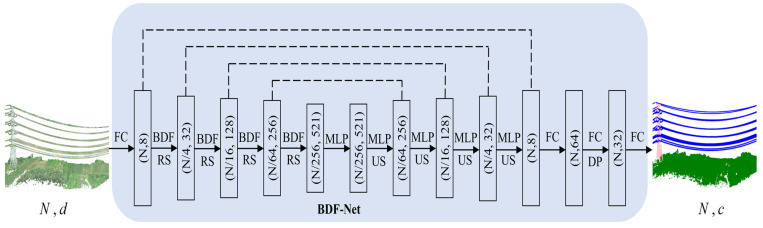
BDF-Net network architecture. BDF: Bilinear Distance Feature, RS: Random Sampling, MLP: Shared MLP, US: Up Sampling, DP: Dropout, FC: Fully Connected layers.

In this section, we introduce a method called BDF-Net that improves RandLA-Net, a point cloud semantic segmentation method specifically designed for power transmission line corridor scenes. The main focus of this method is to infer the spatial geometric features of each point cloud and assign them to the corresponding target object category. Figure 2 illustrates the architecture of BDF-Net. The network takes as input a point cloud of size N×d, which N represents the total number of points and d represents the input feature dimension. First, a fully connected layer is used to extract features for each point, with a dimension set to 8. Then, random sampling is employed for downsampling, and the BDF module is embedded to learn contextual features on the point cloud. This is followed by four cascaded encoder layers for feature encoding, reducing the number of points from N to 1/256 of the original while increasing the feature dimension from 8 to 512. Next, four decoder layers are used to decode the features, employing nearest neighbor interpolation for upsampling and connecting them with intermediate feature maps of the same dimension from the downsampling process through skip connections. Information fusion and representation are performed using MLP. Finally, three contiguous fully connected layers are used to integrate and predict the features. The output is a predicted result of size N×c, which c represents the number of categories.

**Figure 3:**
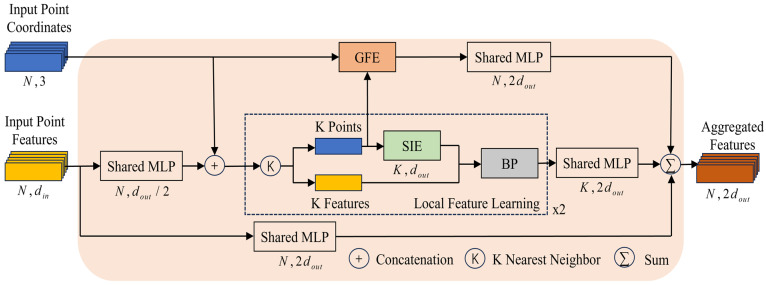
The architecture of BDF modules. The process of point cloud feature extraction is demonstrated.

The detailed structure of the BDF module is shown in Figure 3. It takes as input the point cloud coordinate information and point cloud feature information, using these two pieces of information to learn both the global features and local features of the point cloud. The global features of the point cloud are extracted from the point cloud coordinate information by the GFE (Section 3.3) block to obtain the global characteristics of the point cloud. The local features are learned by the SIE (Section 3.1) block and the BP (Section 3.2) block. The point cloud coordinates and features are aggregated, and through the K-nearest neighbor algorithm, important coordinate information and feature information are selected. The selected coordinate information is then input into the SIE block to learn the local spatial geometric features and, after combining with the selected feature information, it is input into the BP block for bilinear pooling processing, resulting in the local features of the point cloud. The global features, local features, and point cloud feature information are combined to output the aggregated feature representation.

### 3.1. Spatial Information Encoding Block

**Figure 4:**
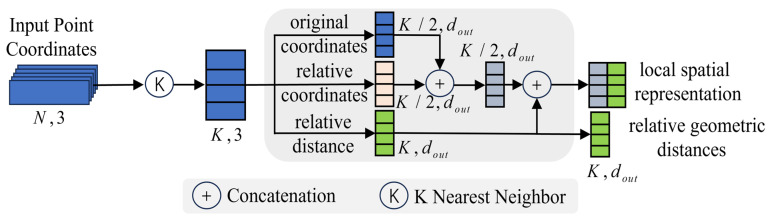
The architecture of the Spatial Information Encoding block.

In real power transmission line corridor scenes, objects belonging to different categories exhibit distinctly different spatial and geometric distributions. For instance, the linear shape of powerlines contrasts sharply with the tower-like structure of pylons. This indicates that features learned from the input point cloud are sensitive to both geometry and spatial information, which can enhance the network’s segmentation performance. To address this, this paper proposes a Spatial Information Encoding block to learn local features of the point cloud, using the Cartesian coordinate system to represent feature information. The architecture of SIE is illustrated in Figure 4.

As seen in Figure 4, the three-dimensional point cloud coordinate information is input into the SIE block for processing. The output consists of local spatial information representation and relative geometric distances.

The SIE consists of the following steps:

Process the original point cloud coordinates. First, the point cloud coordinates are directly used as input, which is represented as a collection of 3D point clouds $\left\{P_{i}|i=1,2,\ldots ,n\right\}$. For the point $P_{i}$, the K-Nearest Neighbors algorithm is employed to obtain a set of k nearest neighboring points $\left\{P_{i}^{1},P_{i}^{2},\ldots ,P_{i}^{k},\ldots ,P_{i}^{K}\right\}$. The KNN algorithm is based on Euclidean distance. The initial local feature representation is denoted as $\left(Doc_{i},Drc_{i}^{k},Dis_{i}^{k}\right)$.

(1) $$Doc_{i}=\left(x_{i},y_{i},z_{i}\right)$$

(2) $$Drc_{i}^{k}=\left(x_{i}-x_{i}^{k},y_{i}-y_{i}^{k},z_{i}-z_{i}^{k}\right)$$

(3) $$Dis_{i}^{k}=\left\|Doc_{i}-Drc_{i}^{k}\right\|$$

where $Doc_{i}$ represents the original input point coordinates, $Drc_{i}^{k}$ denotes the relative coordinates between the input point and its neighboring points, and $Dis_{i}^{k}$ represents the relative geometric distances in the Cartesian coordinate system.

Compute the local position representation. This paper randomly selects K/2 instances of $Doc_{i}$ and $Drc_{i}^{k}$, combining each point’s original position coordinates with its relative position coordinates to its neighbors. These positional features d are then extracted through a convolutional layer.

(4) $$Dlr_{i}^{k}=conv2d\left(concat\left(Doc_{i},Drc_{i}^{k}\right),K/2\right)$$

where concat represents the concatenation of position coordinates along the same dimension, and conv2d indicates that the concatenated information will undergo convolution processing with a kernel size of 1×1.

This processing method can reduce the amount of data to be processed, thereby increasing computational speed. Simultaneously, it enriches the depth information of the data, effectively reflecting the local neighborhood positional characteristics of the point cloud.

Spatial information encoding. The spatial information in the Cartesian coordinate system includes the local position feature representation $Dlr_{i}^{k}$ of neighboring points and the relative Euclidean geometric distance $Dis_{i}^{k}$. The local spatial information is the concatenated encoding of $Dlr_{i}^{k}$ and $Dis_{i}^{k}$.

(5) $$Dlsr_{i}^{k}=MLP\left(concat\left(Dlr_{i}^{k},Dis_{i}^{k}\right)\right)$$

where $Dlsr_{i}^{k}$ represents the local spatial feature information for each point, MLP denotes a convolutional layer with a kernel size of 1×1, and concat indicates the concatenation of each point’s position features and relative geometric distance features along the same dimension.

### 3.2. Bilinear Pooling Block

**Figure 5:**
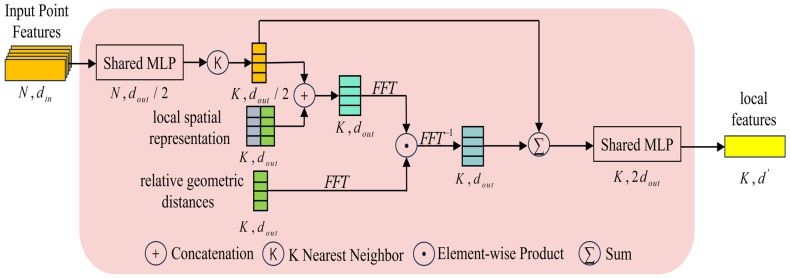
The architecture of the Bilinear Pooling Block.

Inspired by Lin et al. [26], who proposed Bilinear Models for modeling local feature interactions in image recognition tasks for end-to-end fine-grained classification, this paper introduces a Bilinear Pooling block to better learn local contextual information and measure the correlation differences between points. The architectural design of this block is illustrated in Figure 5.

As shown in Figure 5, the BP block has three inputs—input point features, local spatial representation, and relative geometric distances. It performs bilinear pooling on the input feature information, with the output being the local features of the point cloud.

The BP consists of the following steps:

Compute spatial feature representation. First, this paper applies shared MLP processing to the input point features $f_{i}$, reducing the output channel number to half of the input feature channels, where $f_{i}$ represents the vector of feature channels (such as color, normal, etc.) for the ith point. For the point feature $f_{i}$, the KNN algorithm is used to obtain a set of K nearest neighboring points $\left\{f_{i}^{1},f_{i}^{2},\ldots ,f_{i}^{k},\ldots ,f_{i}^{K}\right\}$. The local spatial representation output from SIE is concatenated with the neighborhood point features to generate a spatial feature representation $Dsp_{i}^{k}$.

Calculate the bilinear features. Next, this paper takes the outer product of vectors $Dsp_{i}^{k}$ and $Dis_{i}^{k}$, bilinear pooling allows all elements of the two vectors to interact multiplicatively, thereby deepening the geometric features of the point cloud. However, this leads to an exponential increase in the number of learning parameters, which would result in a significant increase in computation time and memory consumption. Therefore, this paper needs to avoid direct computation of the outer product. Based on the research by Pham et al. [27], it is known that the tensor sketch of the outer product of two vectors can be equivalent to the convolution of two tensor sketches, where the convolution can be replaced by polynomial multiplication using Fast Fourier Transform. The paper then applies the Inverse Fast Fourier Transform to obtain the interacting bilinear features $Dbp_{i}^{k}$.

(6) $$Dbp_{i}^{k}=FFT^{-1}\left(FFT\left(Dsp_{i}^{k}\right)⊙FFT\left(Dis_{i}^{k}\right)\right)$$

where FFT represents the Fast Fourier Transform (FFT), $FFT^{-1}$ denotes the Inverse Fast Fourier Transform and ⊙ represents the element-wise product. Using the element-wise product allows for an easier expansion of multiple features while preserving the interconnections between features.

Obtain local features. Finally, this paper fuses and concatenates the bilinear features with the point cloud features and processes them through shared MLP as the output of this block, obtaining the local feature representation $Dlf_{i}^{k}$.

(7) $$Dlf_{i}^{k}=MLP\left(\sum \left(f_{i}^{k},Dbp_{i}^{k},Dis_{i}^{k}\right)\right)$$

where Σ indicates that the point cloud features are summed and calculated, MLP denotes a convolutional layer with a kernel size of 1×1.

### 3.3. Global Feature Extraction Block

**Figure 6:**
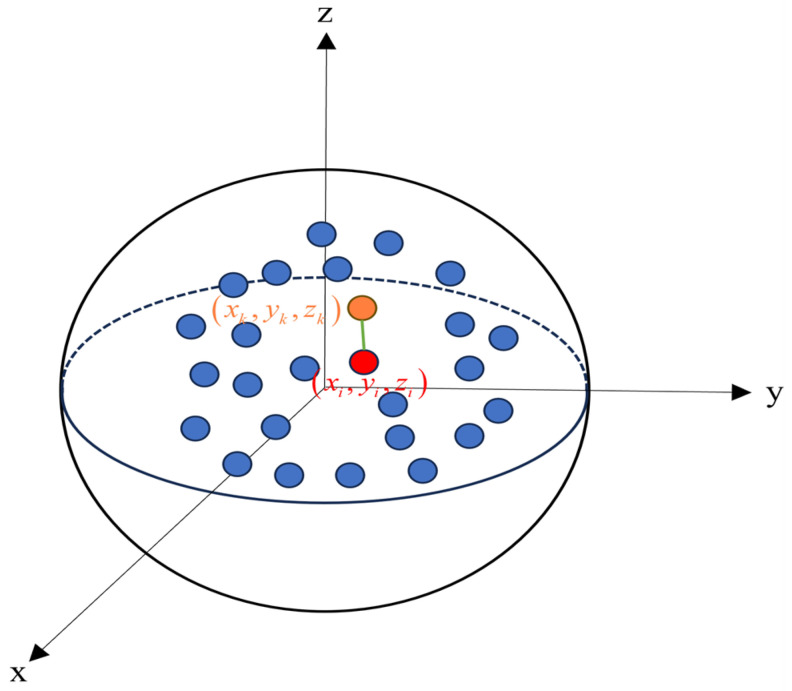
The architecture of the Global Feature Extraction Block. $\left(x_{i},y_{i},z_{i}\right)$ represents the 3D coordinate information of $P_{i}$, $\left(x_{k},y_{k},z_{k}\right)$ represents the 3D coordinate information of the neighborhood point of $P_{i}$.

While local feature representation describes the contextual information of the point cloud within the neighborhood range, it lacks the depiction of global features for semantic segmentation of the entire scene. To increase the receptive field of each point and express point cloud features more comprehensively, this paper proposes a Global Feature Extraction (GFE) block. The architectural design of GFE is illustrated in Figure 6.

The GFE consists of the following steps:

Calculate the global positional features representation. As shown in Figure 6, this paper utilizes both local and global positional features of point clouds. Global positional features are represented as ratios of point positions and relative positions, as this approach is insensitive to positional variations while effectively capturing relative distances between points. This ratio is highly inclusive of point positions, where a value close to 1 signifies close spatial proximity between two points.

(8) $$R_{i}=d_{i}/d_{k}$$

where $d_{i}$ represents the positional information $\left(x_{i},y_{i},z_{i}\right)$ of $P_{i}$ and $d_{k}$ represents the neighborhood relative positional information $\left(x_{k},y_{k},z_{k}\right)$ of $P_{i}$.

Calculate global feature representation. The global features in this work are obtained by concatenating the global position features with the local neighborhood position features and then processing them through a shared MLP, resulting in a global feature representation.

## 4. Results and Analysis

### 4.1. Production of Datasets

Since publicly available LiDAR point cloud datasets are not suitable for power transmission line corridor scenes, this paper collected and constructed a point cloud dataset specifically for power transmission line corridors to evaluate the performance of the proposed method. This dataset is named Power Transmission Line Corridor Point Cloud Dataset (PPCD). The collection site is located at a 550 kV power transmission line in Shenyang, Liaoning Province. A total of 54 spans were collected, with each span approximately 350 m long, resulting in a total length of 18.9 km of power transmission line corridor point cloud data. The PPCD was collected by the DJI Warp and Weft M300 RTK drone equipped with an L1 laser scanning lens. The specific parameters include a flight altitude of 100 m, flight speed of 8 m/s, sampling frequency of 124 kHz, echo mode set to triple echo, point cloud density of 748 points/m^2^, Ground Sampling Distance of 3.27 cm/pixel, scanning mode set to non-repeated scanning, Field of View of 70.4° × 77.2°, true color coloring mode enabled, pixel resolution of 20 million, and using WGS 84 as the RTK reference system.

**Figure 7:**
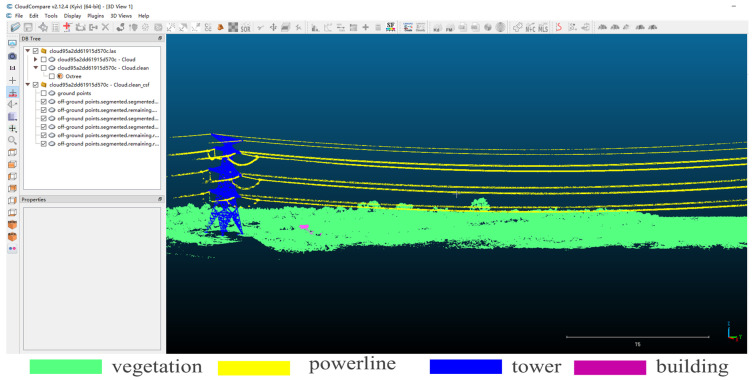
Dataset labeling. Manual annotation using CloudCompare software.

Using DJI Terra V3.4.4 software (https://www.dji.com), the raw collected data were processed for three-dimensional point cloud reconstruction. The specific settings included a point cloud density percentage of 100%, selecting the scene as point cloud processing, a valid point cloud distance of 250 m, enabling point cloud accuracy optimization and point cloud smoothing mode, choosing the known coordinate system WGS 84 for the output coordinate system, and setting the elevation to Default. Then, the results were merged and saved in LAS format. The three-dimensionally reconstructed LAS point cloud was imported into CloudCompare V2.12.4 software (https://www.cloudcompare.org) where statistical filtering was applied to remove outliers, with a setting of 10 neighboring points. Based on the research needs, the point cloud data for the power transmission line corridors was manually annotated, comprising a total of six areas and 70 power transmission line corridor scenes, segmented into four semantic elements—power lines, vegetation, poles/towers, and buildings. The three-dimensional point cloud data include three-dimensional coordinates, RGB information, and semantic labels. Figure 7 shows the labeling information of the dataset.

In order to verify the method of this paper, the data in PPCD are divided into a training set and a test set according to the ratio of 6:1. The training set includes 60 transmission line corridor scenarios with 301 transmission lines, 311 vegetation, 29 towers, and 90 buildings. The test set includes 10 transmission line corridor scenarios with 32 transmission lines, 67 vegetation, 5 towers, and 48 buildings, as shown in Table 1.

Through these precise parameter settings and data acquisition methods, the PPCD dataset can provide high-precision and high-resolution transmission line corridor scenario data, which provides a solid data foundation for related research.

### 4.2. Experimental Settings

The experiment was conducted on a server running Ubuntu 22.04, equipped with an AMD Ryzen Threadripper PRO 3975WX 32-Core processor (AMD, Santa Clara, CA, USA) clocked at 3.50 GHz and a single NVIDIA RTX A4000 GPU with 16 GB of VRAM (NVIDIA, Santa Clara, CA, USA). The software environment consisted of Python 3.6, TensorFlow 2.6, and CUDA 11.7.

The network was trained for 100 epochs with an initial learning rate of 0.01, which was reduced by 5% after each epoch. The batch size was set to four, and the number of neighbors K in the KNN algorithm was set to 16. The Adam optimizer was employed for training. During the training process, approximately 105 points were sampled from each training point cloud, while the entire original point cloud was used for testing. In the experiments, each point cloud contained three-dimensional coordinates and RGB information. Overall Accuracy (OA), mean Intersection over Union (mIoU), and Mean Accuracy (mAcc) are used as evaluation indexes.

### 4.3. Analysis of Semantic Segmentation Results

In this section, the proposed BDF-Net method was evaluated on the power transmission corridor dataset PPCD. To assess the semantic segmentation performance, Areas 1–4 and Area 6 were used as the training set, while Area 5 served as the test set.

**Figure 8:**
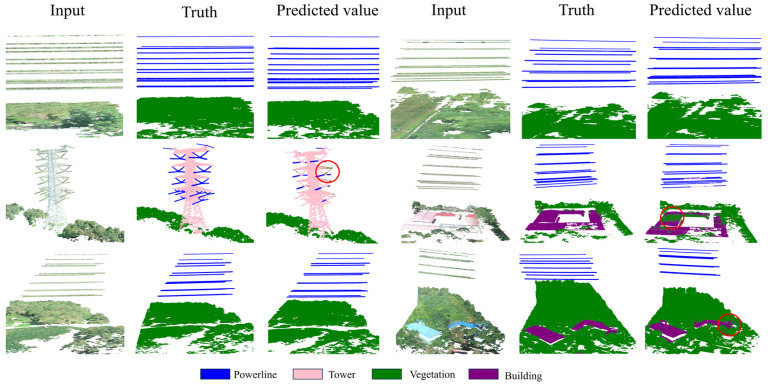
A visualization of the prediction results is shown. Input represents the original point cloud input, Truth represents the true value of scene segmentation, and Predicted value represents the predicted value of network segmentation in this paper. The red circles indicate the location of some misjudgments.

For the task of tree barrier detection in power transmission lines, accurate segmentation of powerlines is crucial, as it is necessary for calculating the sag of transmission lines. Figure 8 presents visualization results for partial scenes, encompassing all categories. The results demonstrate that the proposed method performs well in segmenting powerline and vegetation categories. However, some misclassification issues persist. For instance, there are inaccuracies in identifying the connection points between powerlines and towers, where parts that should be classified as powerlines are mistakenly labeled as towers. Additionally, there are instances where the tops of buildings are misclassified as vegetation.

To visually demonstrate the effectiveness of the proposed BDF-Net method in point cloud semantic segmentation, this paper compares it with state-of-the-art methods such as PointNet++ [10], RandLA-Net [14], MSIDA-Net [19], and SPoTr [28]. The proposed method performs well, outperforming the state-of-the-art methods in three metrics—OA, mIoU, and mAcc. Notably, it achieves the highest segmentation accuracy of 77.48% in mIoU and performs optimally in two categories, including powerline and tower, as shown in Table 2. Compared to RandLA-Net [14], BDF-Net not only improves OA by 3.03% but also achieves a 16.23% gain in mIoU. In comparison with SPoTr [28], BDF-Net shows a 5.8% improvement in mIoU for the powerline category. Compared to MSIDA-Net [19], the BDF-Net in this paper achieves a 19.98% improvement in the building category. When compared to PointNet++ [10], the proposed BDF-Net achieves a 7.67% gain in mIoU for the tower category. It should be noted that in the PPCD dataset, the BDF-Net proposed in this paper ranks second in the vegetation category, slightly lower than PointNet++ [10], indicating that it can still maintain high segmentation performance for large-scale and densely distributed vegetation areas. In terms of classification performance on buildings, BDF-Net is close to SPoTr [28], with a difference of 0.89%. This is because most objects in the building category are composed of planar shapes, including horizontal and vertical planes, and some buildings are covered by vegetation, making the classification task for the building category more difficult. However, compared to other methods, there is a significant improvement. The above results demonstrate the effectiveness of the BDF block, especially the BP block, which can fuse spatial geometric features and enhance the network’s understanding of local details.

**Figure 9:**
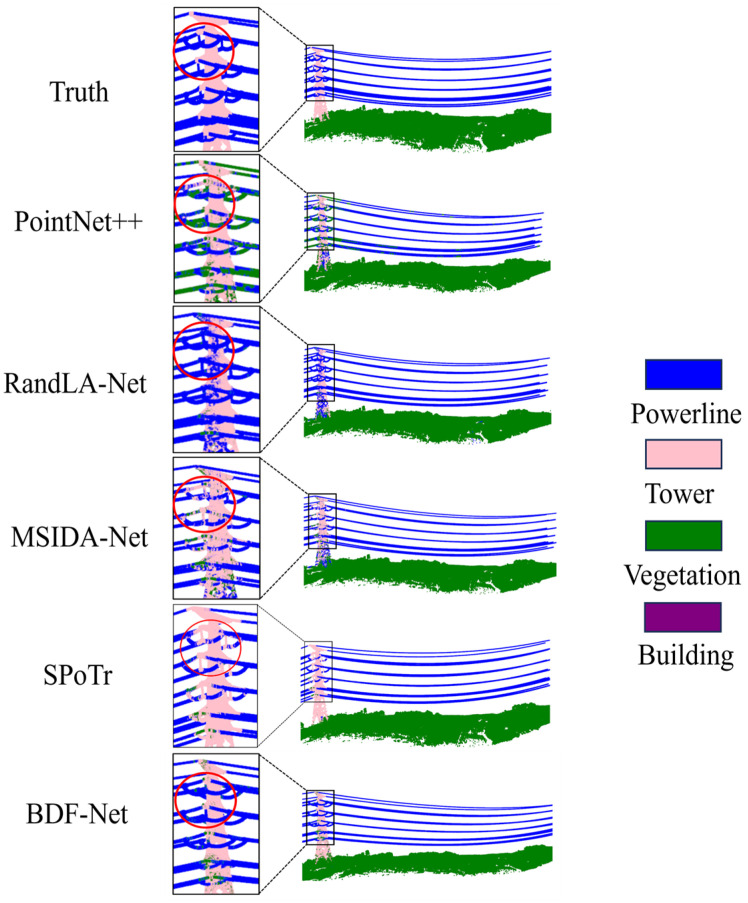
Visualization of segmentation results compared to state-of-the-art methods. The red circles indicate the segmentation results of different methods at the same location.

The comparison of the segmentation results in Figure 9 reveals that significant misclassification phenomena are mainly concentrated in the connection areas between powerlines and towers. This misclassification phenomenon is attributed to the high similarity of the structures at the two connection points, especially the geometric features exhibited by the tips on both sides of the tower, which are quite similar to the power lines thus posing challenges for accurate object boundary segmentation. Although most current comparative methods show obvious limitations when dealing with such areas, the BDF-Net model proposed in this paper demonstrates excellent performance in these key areas. This outstanding performance fully validates the unique advantages and effectiveness of the BDF block in feature aggregation and fusion. In the future, it is possible to further study the chain structure characteristics of power lines, further distinguish them from towers, and divide the transmission line corridor categories in more detail to help improve segmentation accuracy.

### 4.4. Temporal and Spatial Complexity Analysis

**Figure 10:**
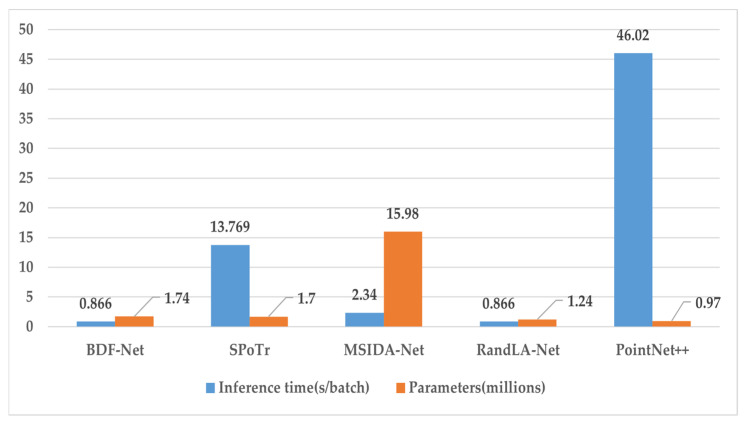
Comparison of inference time and model size for different methods.

In this section, we systematically evaluate the overall efficiency of BDF-Net in performing semantic segmentation on the PPCD dataset, particularly in terms of model inference time and parameter count. For a fair comparison, we input the same number of points (40960) into the network and obtained the model inference time of the proposed BDF-Net and other state-of-the-art methods [10,14,19,28] on the same dataset at Step 700. Additionally, we assessed the model size and reported the parameter count for each network. Figure 10 intuitively illustrates the time consumption and model size of different methods. It can be observed that PointNet++ [10] exhibits the smallest parameter count with its lightweight network design; however, due to its high memory demand for sampling methods, its time efficiency in processing point cloud data is relatively low. RandLA-Net [14] benefits from random sampling and the use of dilated residual structures, which greatly reduces time consumption despite increasing the parameter count. Although MSIDA-Net [19] improves model performance by fusing feature information from multiple coordinate systems, it also leads to a significant increase in network parameter count and a decrease in time efficiency, with its processing time reaching three times that of RandLA-Net [14]. SPoTr [28], by utilizing local self-attention and global cross-attention based on self-positioned points for feature extraction, enhances feature extraction capability but also significantly increases memory consumption thus prolonging network processing time. The proposed BDF-Net, based on random sampling methods, achieves deep understanding and efficient processing of point cloud data by fusing geometric features and spatial features. Despite a slight increase in parameter count, the inference time does not increase, demonstrating that BDF-Net can effectively extract semantic information from point cloud data while maintaining efficient inference speed. Specifically, BDF-Net can achieve a fast inference of 0.866 s on each batch of point cloud data, providing an efficient and accurate solution for semantic segmentation in power transmission line corridor scenes.

### 4.5. Ablation Study

#### 4.5.1. Ablation Study on Activation Functions

**Figure 11:**
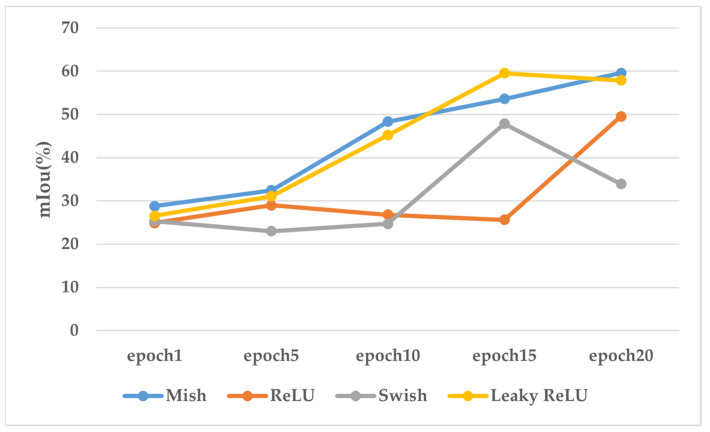
Performance of different activation functions in a segmentation task.

In this section, the effects of four activation functions, including Mish, ReLU, Swish, and Leaky ReLU, were evaluated, where each activation function was tested with the same input point cloud for 20 epochs. The mIoU was recorded at the 1st, 5th, 10th, 15th, and 20th epochs. The training accuracy and stability of each activation function were summarized for comparison, as shown in Figure 11. The results indicate that Mish, ReLU, and Leaky ReLU achieved satisfactory performance within 20 epochs, except for Swish. However, Leaky ReLU reached its peak mIoU at the 15th epoch, followed by significant fluctuations and a sharp decline. Similarly, Swish and ReLU exhibited instability with sudden increases or decreases in training accuracy. In contrast, Mish not only achieved good training accuracy but also demonstrated a stable upward trend overall. Although ReLU only requires a single threshold to obtain activation values, it suffers from the “dead ReLU” phenomenon in negative regions. Leaky ReLU attempts to address this issue by initializing neurons with a small value (e.g., 0.01) but still faces the problem of non-zero mean outputs, leading to biased weight training and affecting accuracy. Swish, while lacking a saturation zone and possessing some smooth progressiveness, does not provide sufficient balance to support effective network learning and inference, given the large span of 3D coordinates in the PPCD dataset. Mish offers better smoothness and allows slight negative values, promoting effective information propagation in neural networks. It maintains continuity and stability during gradient transmission, reducing the occurrence of vanishing or exploding gradients, thereby enhancing model accuracy and generalization capabilities. Consequently, the Mish activation function was employed in BDF-Net.

#### 4.5.2. Ablation Study on BP Block

In this section, we delve into the impact of different pooling methods in the BP block of BDF-Net on semantic segmentation performance. To systematically evaluate the effects of these pooling methods, we selected five different pooling strategies, including Max Pooling, Attention Pooling, Mean Pooling, Mixed Pooling, and the proposed Bilinear Pooling. We conducted comprehensive ablation studies on our carefully constructed power transmission line corridor point cloud dataset PPCD and reported the comparative results in Table 3. Our benchmark network employs Attention Pooling, which adaptively learns local features through an attention mechanism, achieving a mIoU of 61.25%. However, considering the relatively simple object categories in the power transmission line corridor scene, we attempted to use a more concise Max Pooling to fuse spatial features and geometric features. Nevertheless, Max Pooling has limitations in preserving position information, resulting in a 0.05% decrease in accuracy, failing to meet our expectations. Next, we evaluated the performance of Mean Pooling. Although Mean Pooling can retain more background information, it has a lower sensitivity to texture features thus exhibiting a mediocre performance in accuracy improvement. Similarly, Mixed Pooling is more prominent in preserving feature information, but due to its high sensitivity to parameter selection, its improvement in network performance is not significant. Finally, we introduced Bilinear Pooling, which fuses spatial features and geometric features through bilinear interaction. Experimental results demonstrate that Bilinear Pooling improves accuracy by 1.44%, validating its superiority in feature information fusion and utilization. The effectiveness of bilinear pooling lies in its ability to more comprehensively fuse feature information, expand information depth, and refine feature representation in local regions, thereby significantly enhancing the learning capability of the network. In summary, through an in-depth comparison and analysis of different pooling methods, we have verified the superiority of Bilinear Pooling in the semantic segmentation task of power transmission line corridor scenes and provided valuable references for future research.

#### 4.5.3. Ablation Study on BDF Block

In this section, we conducted an ablation study on the BDF block, and the results are shown in Table 4. Ours1 indicates that no feature extraction block is used, which also leads to poor test results. Ours2 indicates that only the SIE block is used. We can see that compared to Ours1, the mIoU is improved by 9.07%, highlighting the effectiveness of the SIE block in local feature extraction and enhancing the network’s learning ability. This result demonstrates that the SIE block can effectively capture local details of point clouds and provide richer local feature representations for the network. Ours3 indicates that only the BP block is used, and although its mIoU is only improved by 1.92%, it still proves the positive role of the BP block in the network structure. The BP block, through its unique mechanism, promotes the network’s understanding and modeling of specific structures in point clouds. Ours4 indicates that only the GFE block is used, and compared to Ours1, the mIoU is improved by 4.47%. This result significantly demonstrates the potential of the GFE block in enhancing the network’s ability to understand global features. The GFE block, by capturing the global contextual information of the image, helps the network better understand the role and relationship of local features in the global structure. To further explore the synergistic effects between different blocks, we combine the SIE, BP, and GFE blocks in pairs in the Ours5, Ours6, and Ours7 models, respectively. These combination models all exhibit excellent performance in network accuracy, achieving varying degrees of improvement. In particular, the combination of the SIE and BP blocks has a remarkable enhancement effect on local feature extraction, further improving the network’s understanding of local expressions by aggregating features of neighboring points. Finally, our overall network architecture, BDF-Net, achieves a significant improvement of 16.23% in mIoU by comprehensively integrating the SIE, BP, and GFE blocks. This not only greatly enhances the network’s expressive ability but also significantly improves the network’s learning ability for contextual features. This result indicates that by reasonably designing and combining different feature extraction blocks, we can build more efficient and accurate deep network models to tackle various complex visual tasks.

## 5. Conclusions

In this paper, we propose an improved RAND semantic segmentation method for transmission line corridor scenarios based on airborne LiDAR data, named BDF-Net. The core research focus of this method is on how to efficiently utilize geometric and spatial information in large-scale point cloud data to achieve fine-grained semantic segmentation tasks. The architecture design of BDF-Net mainly consists of the following three blocks: Spatial Information Encoding (SIE), Bilinear Pooling (BP), and Global Feature Extraction (GFE). The SIE and BP blocks work together to learn local features of point cloud data and perform effective feature fusion, thereby significantly enhancing the network’s perception and expression ability of local details. Through the combination of these two blocks, BDF-Net can fully capture the local geometric structures and spatial relationships in point clouds. The GFE block is used to compensate for the lack of global feature mapping, effectively extracting global information of the entire scene and expanding the receptive field of each point, thus helping the network better understand and distinguish objects of different categories. By fusing global and local features, BDF-Net can provide more comprehensive and accurate semantic segmentation results. To validate the effectiveness of BDF-Net, we constructed a point cloud dataset specifically for power transmission line corridor scenes called PPCD. On this dataset, BDF-Net demonstrated excellent performance, achieving an OA of 97.16%, a mIoU of 77.48%, and a mAcc of 87.6%. Compared to the existing state-of-the-art method, RandLA-Net [14], BDF-Net achieved significant performance improvements in all metrics, with increases of 3.03%, 16.23%, and 18.44%, respectively, further proving its effectiveness in real-world application scenarios. Although BDF-Net has achieved remarkable results in the semantic segmentation task of power transmission line corridor scenes, we still believe that there are some directions worth further exploration and improvement. In particular, how to more accurately describe the boundary issues between objects and how to improve the ability to distinguish details at the connections and similarities of different categories will be important directions for future research. We expect that through continuous research and optimization, we can further enhance the performance of BDF-Net and provide more reliable and efficient solutions for semantic segmentation in power transmission line corridor scenes.

## Figures and Tables

**Table 1:** Partitioning of the dataset.

Dataset	Powerline	Vegetation	Tower	Building
train	301	311	29	90
test	32	67	5	48

**Table 2:** Comparison with state-of-the-art methods.

Methods	OA(%)	mIoU (%)	mAcc (%)	Powerline	Vegetation	Tower	Building
PointNet++ [10]	92.6	64.52	72.4	62.4	99.5	77.7	18.48
RandLA-Net [14]	94.13	61.25	69.16	61.63	95.11	65.86	22.4
MSIDA-Net [19]	96.18	64.02	73.97	63.61	96.12	67.02	29.34
SPoTr [28]	95.68	75.97	85.24	71.74	96.63	85.3	50.21
BDF-Net (ours)	97.16	77.48	87.6	77.54	97.69	85.37	49.32

**Table 3:** Results of ablation experiments with the BP block.

Methods	mIoU (%)
Max Pooling	61.20
Attention Pooling	61.25
Mean Pooling	61.07
Mixed Pooling	61.69
Bilinear Pooling(ours)	63.17

**Table 4:** Ablation experiments of the BDF block.

Methods	SIE	BP	GFE	mIoU (%)
Ours1				61.25
Ours2	√			70.32
Ours3		√		63.17
Ours4			√	65.62
Ours5	√	√		76.23
Ours6	√		√	76.04
Ours7		√	√	74.31
BDF-Net	√	√	√	77.48

## Data Availability

The raw data supporting the conclusions of this article will be made available by the authors on request.
